# Collodium, or Collodion

**Published:** 1848-11

**Authors:** 


### ACADEMY OE MEDICINE.

*Collodium, or Collodion.*—M. Souberian mentioned a new method of preparation of this adhesive substance ; his preparation yielded a purer collodium, and better calculated for rendering tissues waterproof.

E. Malgaigne stated, that for surgical purposes, a certain degree of impurity of the collodium is necessary; he preferred that obtained by the process pointed out by M. Mialhe. The following is the plan followed by that chemist :—

Pure gun-cotton, that which left no residue whatever after deflagration, was not proper for the preparation of collodium. A special gun-cotton should be used, obtained by the reaction of sulphuric acid and nitre upon cotton. Some precautions were indispensable: ʒv. of cotton, ℥xiv. of sulphuric acid, and ℥x. of nitre, were the proportions of the various elements. The nitre and sulphur should be mixed in a china capsule, and, the cotton being added, the mixture should, during a space of three minutes, be agitated with glass sticks. After being well dried, the gun-cotton thus obtained was not very pure; it always contained sulphuric acid, but it had acquired the property of dissolving readily in ether, and, better still, in ether and alcohol. The formula was the following;—

℞. Sulphuric gun-cotton, ʒij. rectified sulphuric ether, ℥iv.; rectified spirit, ʒij.

It was absolutely necessary that the parts upon which the adhesive liquid was applied should be preserved from all humidity until the ether had completely evaporated.

